# Dementia palliative care education and training for healthcare professionals: A scoping review protocol

**DOI:** 10.12688/hrbopenres.13486.2

**Published:** 2023-01-20

**Authors:** William Hutch, Trish O' Sullivan, Tony Foley

**Affiliations:** 1 Department of General Practice, University College Cork, Cork, Ireland; 2 Clinical Fellow, Irish College of General Practitioners (ICGP), Irish College of General Practitioners, Dublin, D02 XR68, Ireland; 3 Discipline of Physiotherapy, School of Clinical Therapies, University College Cork, Cork, Ireland

**Keywords:** Dementia, Palliative Care, Education, Training, Healthcare Professionals

## Abstract

**Background: **Global mortality rates from dementia continue to rise.  Evidence suggests that there is limited provision of palliative care for people with dementia and this is a cause of grave concern. The coronavirus disease 2019 (COVID-19) pandemic has further exposed the inequalities of care for this vulnerable population. Proactive palliative care, delivered by multidisciplinary healthcare professionals (HCPs), can offer significant benefits to people with dementia.  However, little is known about the components of effective education and training for HCPs who care for people with advancing dementia at end of life.

**Objective:** The aim of this scoping review is to identify effective education and training interventions for HCPs, who care for people with advanced dementia approaching end of life.

**Inclusion criteria:** Studies that used a palliative care educational intervention for HCPs working with patients with dementia will be included. Studies that explore undergraduate or postgraduate education and training in dementia palliative care for HCPs will be included.  Study designs such as quantitative, qualitative, mixed method studies, and case studies will be included.

**Methods:** The Joanna Briggs Institute (JBI) methodology for scoping reviews will be used for this review. The following databases will be searched: CINAHL, ERIC, Medline, SocIndex, PsycINFO. In addition, grey literature searches will be limited to the first 100 searches using Google Scholar and Open-Grey. Study selection will involve the reviewer screening titles and abstracts. Then, two independent reviewers will further assess the studies in full for those that meet the inclusion criteria. In line with the JBI framework, data will be extracted using a draft data extraction tool. This will facilitate a chronological narrative synthesis of results in line with the study’s overall aim to identify effective education and training interventions for HCPs, who care for patients with dementia, nearing end of life.

## Introduction

Dementia can be defined as an insidious, chronic progressive disability of an individual’s cognition and physical characteristics, leading to progressive impairment of cognitive functioning, particularly in the domains of memory, comprehension, language, orientation and judgement ^1, 2^. Dementia is the seventh leading cause of death in the world ^3^. It is estimated that 55 million people have dementia worldwide at present and this is set to increase to 139 million by 2050 ^3^. Additionally, global mortality rates from dementia have increased, that is, between 2000 and 2019, the number of deaths from Alzheimer’s disease have more than doubled, increasing by 145.2% based on death certificates’ records ^1^. 

The coronavirus disease 2019 (COVID-19) pandemic has further exposed limitations of care for persons with dementia ^4^. There were at least 42,000 more deaths from Alzheimer’s disease and other dementias in 2020 compared with the average of the five years before 2020 ^5^. Moreover, for individuals aged over 85 years who died of COVID-19, dementia was listed in up to 20% of causes as the cause of death ^6^. The risk factors for dementia in general are also the major risk factors for poor outcomes which COVID-19 ^7, 8^. The pandemic has also led to concerns about a proliferation in the use of anticipatory care that has not been researched or evaluated, for example escalated antipsychotic use and the rationing of care for frail persons including those with dementia, in contravention to their human rights ^9^. In addition, the lack of appropriate palliative care for individuals with dementia during the COVID-19 pandemic is of grave concern, as many patients have not been able to adequately receive care ^10^.

Proactive palliative care in persons with dementia is favoured by caregivers and clinicians and offers benefits throughout the course of the illness, from diagnosis to the terminal phase ^11, 12^. Palliative care can be defined as: “an approach to care that improves the quality-of-life of patients and their families facing problems associated with life-threatening illness, through the prevention and relief of suffering by means of early identification and impeccable assessment and treatment of pain and other problems, physical, psychosocial and spiritual” ^13^. Persons with dementia are not often identified as having palliative care needs, for example, it is recognised that they may suffer in the advanced stages with symptoms such as pain, breathlessness, eating problems, neuropsychiatric symptoms and as a result this often leads to delays in them receiving palliative care ^14^. Moreover, timely recognition of these symptoms for individuals with dementia approaching end of life is important to ensure that they receive the right amount of care at the right time ^15^.

The Irish National Dementia Strategy ^16^ highlights that training and education for healthcare professionals in dementia palliative care is one of its primary goals. However, education and training for healthcare professionals (HCPs) working with individuals with advanced dementia approaching end of life is often limited. Internationally, the US Department of Health and Human Services ^17^ highlight that healthcare skills and knowledge to provide effective care for people with dementia is lacking. This is further demonstrated by Eriksson and Saveman by identifying that poor staff skills, knowledge and attitudes are reported to contribute to low standards of care of Persons with Dementia ^18^.

While education and training of HCP has been advocated, there is limited evidence regarding the effective components of dementia palliative care educational interventions or whether these interventions lead to positive outcomes. Educational interventions need to move beyond simple dissemination of knowledge to the actual application and use of knowledge. A preliminary search of the Medline (via EBSCO) and CINAHL databases did not find any scoping reviews for dementia palliative care education and training for HCPs. In addition, the PROSPERO database was searched and did not identify any similar review protocols. Due to this limited evidence, our aims were broad which was more suited for a scoping review, rather than a systematic review which would be to sum up the best available research on a specific question ^19, 20^.

### Aim

This scoping review aims to identify effective education and training interventions for HCPs, who care for people with advanced dementia approaching end of life.

### Objectives

(1) To identify studies that have used education and training interventions for HCPs in patients with dementia, nearing end of life.
(2) To critically appraise and identify curricular components of effective interventions in this patient population.
(3) To use this critically appraised evidence base, to inform the design of dementia palliative care education and training for HCPs.

### Purpose

To identify and understand effective educational/training interventions to date that are in use by HCPs for Persons with Dementia. To inform the future design of effective dementia education/trainings for HCPs, with the aim of leading to better dementia palliative care for Persons with Dementia.

## Methods

The Joanna Briggs Institute (JBI) methodology will be implemented for this scoping review due to its consideration and effectiveness of primary evidence found in both qualitative and quantitative research ^21^. This scoping review protocol was registered with Open Science Framework on 30 December 2021 (DOI 10.17605/OSF.IO/BWQU7). 

### Inclusion criteria

***Population.*** This review will consider studies that include an education or training intervention for HCPs in the area of dementia palliative care from 01/01/1990 to 30/12/2021 HCPs will be defined based on the World Health Organisation (WHO) guidelines for individuals that work in a health care setting ^22^. These will include medical doctors both generalist and specialist practitioners, nursing professionals including public health nurses, speech and language therapists, occupational therapists, physiotherapists, psychologists, and dietitians. This list is not exhaustive for the purposes of inclusion criteria as any studies that include HCPs that attended training may be considered. Furthermore, studies with participants undertaking education/training in both undergraduate and postgraduate settings will be included. 

***Concept.*** This scoping review protocol is developed to determine the type and extent of educational interventions available for HCPs working with people with dementia palliative care. Therefore any study with a focus on education and training for healthcare professionals will be reviewed and considered. The Cochrane Effective Practice and Organisation of Care Group (EPOC) outline clear definitions of educational interventions such as educational outreach, meetings, audit and feedback and they have reviewed how such interventions lead to behavioural change as well as knowledge translational activities ^23^. Based on the EPOC, the authors will ensure the inclusion of studies that meet the definition of the educational interventions as per their guidelines.

Education for HCPs is multifaceted, comprising of educational philosophy, theory, principles and processes to generate knowledge synthesis, that often takes place in a busy clinical environment to solve complex clinical problems ^23, 24^. Effective education can be measured based on learning outcomes, which are ‘ideally written as specific, measurable, attainable, relevant and ‘time-bound’ ^24^. Traditional education domains include cognitive, psychomotor and behaviours ^24^, however, training in healthcare education includes domains that are more complex because of their clinical context and this is particularly true for patients with dementia approaching end of life. Effective training is the application and mastery of these skill domains which promotes learning ^24^. As dementia care includes multiple domains of clinical, ethical, social and medical issues, this study will include both aspects of education and training for HCPs working with persons with dementia. 

***Context.*** HCPs working with persons with dementia undertake their education and training in a range of settings such as their practice, nursing homes, hospitals, palliative care specialist centres or the patient’s home. As a result, any studies where education and training in any settings or location will be considered in this review.

***Types of studies.*** Qualitative, quantitative, mixed methods studies and observational studies that use an educational or learning intervention for HCPs working persons with dementia in a palliative care setting. Peer review is not a requirement for studies to be included. Case studies will not be included in this scoping review.

### Search strategy

A three-step search strategy will be used for this protocol as outlined by the JBI guidelines ^25^. Step one has already been completed; this included a broad search of CINAHL and Medline (via EBSCO by reviewer WH) which included key words for healthcare professionals, dementia, palliative care and education/ training. Step two; the titles and abstracts were reviewed to ensure key words matched the study criteria ^26, 27^, thus ensuring that there was adequate data available to undertake this review. The search strategy was developed by two reviewers (WH and TF) with the support of a librarian in University College Cork. This search strategy is outlined in Table 1, which outlines the language filters and the date of the search. Step three of the search strategy will include a review of the reference lists of the studies that meet the search criteria. The following data bases will be searched based on guidance by a librarian, these will include CINAHL, ERIC, SocINDEX, Medline, PsycINFO and Cochrane. In addition, grey literature searches will include Google Scholar and Open-Grey.

**Table 1.** Strategy: CINAHL plus full text. Date of search: 09/12/21.

Number	Search Terms	Records Retrieved
# 1	“Healthcare profession*”OR “Social Care Profession*” OR “Speech and Language” Or Nurs* OR “Psycholog*” or Physio* Or “Occupational Ther*” OR GP* OR “general pract*” OR “family practi*” Or “Dieti*” OR “Primary Care”	2,191,385
# 2	Dementia OR Alzheimer* OR “cognitive impairment” OR “memory loss”	127,294
# 3	“Palliative Care” OR “End-of-Life Care” OR “Terminal care” OR “Hospice Care”	69,668
# 4	Education OR Training OR Learning OR Teaching OR Workshop*	1,013,915
# 5	#1 and # 2	50,036
# 6	#2 and #3	2,741
# 7	# 3 and # 4	12,660
# 8	# 1 and # 2 and # 3	1,442
# 9	# 1 and #2 and # 3 and # 4	356

### Study selection

Once the above JBI three step search strategy has been completed, all the relevant and identified citations will be uploaded to EndNote 20. Next, the titles and abstracts will be independently reviewed by two reviewers (WH and TF) using Rayyan software ^27^ for assessment of inclusion criteria. If uncertainty exists, a third reviewer (TOS) will independently assess. The next step will involve screening the full text of selected studies will be screened. Studies that do not meet the inclusion criteria will be excluded. A detailed documentation outlining the search will be reported in a preferred reporting items for systematic reviews and meta analysis (PRISMA) diagram ^28, 29^.

### Data extraction

In line with the JBI guidelines, data extraction will include study characteristics such as author, year, type of study, publication titled, country, clinical settings, participant, purpose, education content, mode of delivery and key findings. The Kirkpatrick’s Framework will be used to critically appraise and report the effectiveness of the educational intervention and to report the outcomes of identified studies to dementia palliative care education for health care professionals ^30, 31^. The Kirkpatrick Framework consists of four levels of hierarchy of assessing education and training and it has been used widely to evaluate educational interventions in healthcare settings ^31^. These four levels are; 1. “ *Reaction* measures the learners’ value they perceive in the educational intervention, 2. *Learning* measures improvements in their knowledge, 3. *Behaviour* measures their capability applied in context, and 4. *Result* measures the impact the training on the target outcome – in this case, patient level outcomes” ^24, 30, 31^. The initial draft data extraction will be independently assessed by two reviewers, and any modifications will be made where necessary. Such modifications will be documented in detail in the scoping review report. The data will be extracted by one reviewer and a second reviewer will determine if any changes need to be made. If any disagreements arise a third independent reviewer will reassess. If any papers do not outline in full their data or if there is missing information, the reviewers will contact the authors to obtain such information. 

### Data presentation

A narrative synthesis of the data in line with our study aims and objectives of this scoping review will be presented in chronological order. This review will highlight the important aspects of education and training for HCPs working with persons with dementia in a palliative care setting. The Kirkpatrick Framework will assess the effectiveness of such educational interventions as outlined above and based on the four levels; various training interventions will be tabulated in their appropriate categories.

## Dissemination of information

This study’s findings will be disseminated via publication in an academic international peer reviewed journal. We also plan to present our findings at national and international conferences targeting HCP, who care for people with advanced dementia approaching end of life. We also aim to disseminate this work through professional bodies such as the Irish College of General Practitioners, the Health Service Executive, and the Dementia Research Network of Ireland.

## Study status

This scoping review is currently at stage one; that is to explore and document the evidence relating to education and training for HCPs by identify effective education and training interventions for HCPs, who care for patients with dementia, nearing end of life.

## Data availability

No data are associated with this article.
