# Supplementary material for: Dementia palliative care education and training for healthcare professionals: A scoping review protocol
Source: HRB Open Res. 2023 Jan 20;5:13. Originally published 2022 Feb 15. [Version 2] doi: 10.12688/hrbopenres.13486.2 (PMC9883599; doi:10.12688/hrbopenres.13486.2)
Supplement: Peer review history

# Peer review: Reviewer response for version 2

**DOI:** 10.21956/hrbopenres.14959.r33375

**Published:** 2023-01-27

**Authors:** Katherine Lawler

**Affiliations:** 1 Wicking Dementia Research and Education Centre, University of Tasmania, Hobart, Tas, Australia

Dear authors,

 Thank you for responding to my comments. I have no further comments to make, and look forward to reading your completed review when it is available.

 Best wishes,

 Kate

Is the study design appropriate for the research question?

Partly

Is the rationale for, and objectives of, the study clearly described?

Yes

Are sufficient details of the methods provided to allow replication by others?

Partly

Are the datasets clearly presented in a useable and accessible format?

Not applicable

Reviewer Expertise:

Clinical care for people with dementia, dementia education for health professionals, systematic and scoping reviews.

I confirm that I have read this submission and believe that I have an appropriate level of expertise to confirm that it is of an acceptable scientific standard.

# Peer review: Reviewer response for version 1

**DOI:** 10.21956/hrbopenres.14707.r31532

**Published:** 2022-04-04

**Authors:** Marieke Perry

**Affiliations:** 1 Department of Geriatric Medicine, Radboudumc Alzheimer Center, Radboud University Medical Center, Nijmegen, The Netherlands

This proposed review addresses a very relevant topic, of which the urgency is clearly described: Palliative care for persons with dementia is limited. Its urgency is underlined by the COVID-19 pandemic. Training of HCPs is needed, but little is known of effective training programs on the topic of proactive palliative care. The introduction could gain from including the white paper of v.d. Steen *et al* (2014) ^1^ which defines relevant elements and issues for palliative dementia care based on a Delphi procedure. 

**Aim:**

 Many palliative care tools and training programs have been developed over the last decade. A review is therefore an adequate design for evidence synthesis.

**Methods:**

 Clearly described according to JBI methods and PRISMA guidelines. The proposed search strategy seems adequate for the design (scoping review). I wonder whether the authors considered including specific proactive palliative care elements as advance care planning (and other elements as defined in the v.d. Steen white paper) in the search strategy, as these may bring more focus to the paper than a broad topic as palliative care. For the categorization of outcomes the Kirkpatrick framework is adequately proposed.

Is the study design appropriate for the research question?

Yes

Is the rationale for, and objectives of, the study clearly described?

Yes

Are sufficient details of the methods provided to allow replication by others?

Yes

Are the datasets clearly presented in a useable and accessible format?

Not applicable

Reviewer Expertise:

Primary care, dementia, advance care planning, interprofessional collaboration

I confirm that I have read this submission and believe that I have an appropriate level of expertise to confirm that it is of an acceptable scientific standard.

## References

[1] : White paper defining optimal palliative care in older people with dementia: a Delphi study and recommendations from the European Association for Palliative Care. *Palliat Med* .2014;28(3) : 10.1177/0269216313493685 197-209 10.1177/0269216313493685 23828874

# Peer review: Reviewer response for version 1

**DOI:** 10.21956/hrbopenres.14707.r31476

**Published:** 2022-04-04

**Authors:** Katherine Lawler

**Affiliations:** 1 Wicking Dementia Research and Education Centre, University of Tasmania, Hobart, Tas, Australia

Thank you for the invitation to comment on this scoping review protocol, which addresses education and training in dementia palliative care, the importance of which has been emphasised during the COVID-19 pandemic. The protocol is well-written, including a comprehensive and appropriate choice of databases and guided by strong methodology through the Joanna Briggs institute.

 The main question I have for the authors is regarding the choice of scoping review methodology rather than systematic review methodology. It seems that the objectives of this review could sit in either camp, and perhaps the choice of scoping review needs to be described a little more clearly. The JBI manual for evidence synthesis has a summary of 'why a scoping review' in section 11.1.1. Of note, systematic reviews may consider qualitative research and quantitative study designs other than randomised controlled trials.

 In addition, please note the following comments:

1. In Australia we are trying to move away from abbreviations such as PwD. I'm not sure if similar guidelines exist in Ireland. This does not necessarily need to be changed in this protocol, but could perhaps be considered when publishing the outcomes. See https://www.dementia.org.au/resources/dementia-language-guidelines 
2. Can you please clarify how the review will be guided by EPOC? Perhaps this will sit better in the methods section.
3. The objectives mention critical appraisal - which would be important to support the review's purpose. However, I am not sure there is any description in the methods about how the included papers will be critically appraised? 
4. Types of studies - please note whether peer review is a requirement or not, in your review.
5. If planning to re-run the search for any purpose, you could consider search terms that will include international terms for some of the allied health professions e.g. physical therapist, speech pathologist. 

Wishing you all the best as you complete this important piece of work.

Is the study design appropriate for the research question?

Partly

Is the rationale for, and objectives of, the study clearly described?

Yes

Are sufficient details of the methods provided to allow replication by others?

Partly

Are the datasets clearly presented in a useable and accessible format?

Not applicable

Reviewer Expertise:

Clinical care for people with dementia, dementia education for health professionals, systematic and scoping reviews.

I confirm that I have read this submission and believe that I have an appropriate level of expertise to confirm that it is of an acceptable scientific standard, however I have significant reservations, as outlined above.

# Response

**Published:** 2023-01-12

**Authors:** William Hutch

**Affiliations:** University College Cork, Ireland

Many thanks for taking the time to review our scoping review protocol and we greatly appreciated your feedback. Please see below our responses to your comments. We hope these are satisfactory. 

 (a) RE: the choice of scoping review methodology rather than systematic review methodology. 

 “The main question I have for the authors is regarding the choice of scoping review methodology rather than systematic review methodology. It seems that the objectives of this review could sit in either camp, and perhaps the choice of scoping review needs to be described a little more clearly. The JBI manual for evidence synthesis has a summary of 'why a scoping review' in section 11.1.1. Of note, systematic reviews may consider qualitative research and quantitative study designs other than randomised controlled trials.” 

 RESPONSE:

 Please see “Introduction” section and we added:

 “Due to this limited evidence, our aims were broad which was more suited for a scoping review, rather than a systematic review which would be to sum up the best available research on a specific question ^19, 20^.”

 19. Arksey H, O’Malley L: Scoping studies: towards a methodological framework. *Int J Soc Res Methodol.* 2005;8(1):19–32. 10.1080/1364557032000119616

 20. Munn Z, Peters MDJ, Stern C, Tufanaru C, McArthur A, Aromataris E. Systematic review or scoping review? Guidance for authors when choosing between a systematic or scoping review approach. BMC Med Res Methodol. 2018 Nov 19;18(1):143. doi: 10.1186/s12874-018-0611-x. 

 In addition, please note the following comments: 

 1. In Australia we are trying to move away from abbreviations such as PwD. I'm not sure if similar guidelines exist in Ireland. This does not necessarily need to be changed in this protocol, but could perhaps be considered when publishing the outcomes. See https://www.dementia.org.au/resources/dementia-language-guidelines 

 RESPONSE: We agree & we have changed “PwD” to “Persons with Dementia” throughout the manuscript

 2. Can you please clarify how the review will be guided by EPOC? Perhaps this will sit better in the methods section.

 RESPONSE: Yes we agree this sits better in the methods section, under the concept heading: 

 The Cochrane Effective Practice and Organisation of Care Group (EPOC) give clear definitions of educational interventions such as educational outreach, meetings, audit and feedback and they have reviewed how such interventions lead to behavioural change as well as knowledge translational activities ^23^. Based on the EPOC, the authors will ensure the inclusion of studies that meet the definition of the educational interventions as per their guidelines. 

 23 Grimshaw JM, Eccles MP, Lavis JN, *et al.*: Knowledge translation of research findings. *Implement Sci.* 2012; **7**: 50.

 3. The objectives mention critical appraisal - which would be important to support the review's purpose. However, I am not sure there is any description in the methods about how the included papers will be critically appraised? 

 RESPONSE: Please see: Methods section; Date Extraction 

 Added “ The Kirkpatrick’s Framework will be used to critically appraise and report the effectiveness of the educational intervention and to report the outcomes of identified studies to dementia palliative care education for health care professionals” 

 4. Types of studies - please note whether peer review is a requirement or not, in your review.

 RESPONSE: Please see: Methods sections, Types of studies;

 “Peer review is not a requirement for studies to be included”

 5. If planning to re-run the search for any purpose, you could consider search terms that will include international terms for some of the allied health professions e.g. physical therapist, speech pathologist. 

 RESPONSE: Yes we agree and we acknowledge this may be a limitation to our search strategy.
